# A Treatise on Those Diseases of Women Which Occasion Leucorrhœa or Other Utero-Vaginal Discharges

**Published:** 1842-10

**Authors:** 


ART. IX.—*Traité des Maladies des Femmes qui déterminent des Fleurs Blanches, des Leucorrhées, ou tout autre Ecoulement Utéro-Vaginal.* Par HENRY BLATIN et V. NIVET.—*Paris*, 1842. 8vo, pp. 632.

*A Treatise on those Diseases of Women which occasion Leucorrhœa or other Utero-vaginal Discharges.* By H. BLATIN and V. NIVET.—*Paris*, 1842. 8vo, pp. 632.

THE authors of this work have proposed to themselves to continue and complete the *Traité du Catarrhe Utérin* of their relative and predecessor, J. B. Blatin. They have accordingly divided the diseases of which they treat into two classes, describing under the head of idiopathic discharges all those affections in which the mucous membrane only of the genital organs is affected, while all cases in which the morbid secretion results from change of structure in some more deeply-seated part, or from ulcerations or the existence of tumours or presence of foreign bodies, are referred to the head of symptomatic discharges. For the second part of the work, with the exception of a chapter on vesicular polypi, which has already appeared in the Archives Générales de Médecine, the writers do not claim any higher merit than that of conscientious compilers. In the former half of the book, which treats of idiopathic leucorrhœa, they do, however, lay claim to greater originality, though acknowledging their obligations to J. B. Blatin as well as to subsequent writers. Their acknowledgments are nevertheless by no means so full as they ought to have been; and after going through the tedious task of collating the two books with each other, we cannot help remarking that the present ought to be regarded rather as a reprint of Blatin's treatise with notes and additions than as an independent work, the result of original observation. The chapters on the nomenclature and classification of the disease, on its causes and on the effects of its suppression, are little else than transcripts from J. B. Blatin's treatise, and in many places the authors have quietly appropriated to themselves the results of their predecessor's learning, quoting cases and opinions, and giving references to the original writers instead of to Blatin, from whose magazine of materials they have borrowed much of their erudition.

Taking the book, however, at the lowest estimate, and regarding it merely as a compilation, it is not without interest, presenting an epitome of the present state of knowledge in France, concerning a class of diseases which have been but comparatively little elucidated in this country. MM. Blatin and Nivet appear to be well acquainted with all the writings of their countrymen on uterine diseases; and notwithstanding a want of precision in their subdivisions and a wearisome prolixity of style, they have produced a work creditable to their diligence and sound practical common sense. When next we meet with them we trust that we may find them in the character of original observers rather than as the retailers of the opinions of others.

